# External Validation of Dementia Prediction Models in Black or African American and White Older Adults: A Population‐Based Study in the United States

**DOI:** 10.1002/alz.087601

**Published:** 2025-01-09

**Authors:** Klodian Dhana, Todd Beck, Xiaoran Liu, Anisa Dhana, Ted K.S. Ng, Pankaja Desai, Denis A Evans, Kumar B Rajan

**Affiliations:** ^1^ Rush University Medical Center, Chicago, IL USA; ^2^ Rush Institute for Healthy Aging, Chicago, IL USA

## Abstract

**Background:**

Identifying people at high risk of dementia allows for timely intervention, potentially slowing cognitive decline and delaying the onset of dementia. We externally validated commonly used risk predictions model for Alzheimer’s dementia in Black American and white older adults.

**Method:**

The study included 2,130 participants with 1,159 Black American and 971 white participants without dementia at baseline from the Chicago Health and Aging Project (CHAP). Based on a recent systematic review and a comparative analysis, four prediction models were selected, including Cardiovascular Risk Factors, Ageing and Dementia (CAIDE), Dementia Risk Score (DRS), Brief Dementia Screening Indicator (BDSI), and Australian National University AD Risk Index (ANU‐ADRI). Using reported scores (i.e., points or beta estimates) from each model, we computed the predicted risk of dementia. The clinical diagnosis of dementia was based on the criteria of the joint working group of NINCDS and ADRDA for probable Alzheimer’s disease. The performance of each prediction model was assessed by the C‐statistic (discrimination ability) and calibration plots (comparing observed vs. expected dementia probabilities).

**Result:**

During 18 years of follow‐up, 275 (23.7%) Black and 167 (17.2%) white older adults developed incident dementia. Of the prediction models evaluated, BDSI had the highest discriminant value for dementia risk with c‐statistics of 0.79 (95%CI 0.74‐0.84) in Black and 0.77 (95%CI 0.70‐0.85) in white individuals. The c‐statistics for ANU‐ADRI were 0.78 (95%CI 0.74‐0.83) in Black and 0.75 (95%CI 0.70‐0.81) in white participants. CAIDE had a lower discriminating power, 0.55 (95%CI 0.51‐0.58) and 0.53 (95%CI 0.48‐0.57), respectively, in Black and white individuals. DRS performed better among individuals aged 65‐79 (c‐statistics: 0.75 in black and 0.66 in white individuals) than 80‐95 (c‐statistics: 0.61 in black and 0.55 in white individuals). The calibration plots showed that prediction models (i.e., DRS and CAIDE) significantly underestimated (i.e., 10 times lower) the risk of Alzheimer’s in both Black and white study participants.

**Conclusion:**

The prediction model assessed in this study underestimated the risk of Alzheimer’s in both Black and white individuals. This underscores the necessity for developing models specific to race to predict the risk of Alzheimer’s dementia more accurately in the population.

